# Urinary Uromodulin Levels and* UMOD* Variants in Black South Africans with Hypertension-Attributed Chronic Kidney Disease

**DOI:** 10.1155/2019/8094049

**Published:** 2019-04-02

**Authors:** Nolubabalo Unati Nqebelele, Caroline Dickens, Therese Dix-Peek, Raquel Duarte, Saraladevi Naicker

**Affiliations:** Department of Internal Medicine, Faculty of Health Sciences, University of the Witwatersrand, Johannesburg, South Africa

## Abstract

Uromodulin, the most abundant protein in urine, is synthesized in the thick ascending loop of Henle and distal convoluted tubules. Patients with chronic kidney disease (CKD) have reduced urinary uromodulin levels secondary to tubular damage. Genome wide association studies identified significant single nucleotide polymorphism (SNP) associations with CKD at the uromodulin (*UMOD) *locus. We examined the association of urinary uromodulin concentrations with CKD and with SNP rs1333226 in the* UMOD* gene. The study included 71 black South Africans with hypertension-attributed CKD with an eGFR ≤ 60ml/min/1.73m^2^, 52 first-degree relatives, and 58 unrelated controls. Urinary uromodulin concentration was measured using Luminex® multiplex kits. After DNA extraction from blood using the Maxwell® automated platform, genotyping of rs13333226 was performed using real-time PCR using TaqMan® genotyping assays. Urinary uromodulin levels were significantly lower in CKD cases compared to both controls and first-degree relatives and correlated negatively with age, serum uric acid, serum creatinine, and systolic BP and positively with CKD-EPI eGFR. For each 1-standard deviation increase in uromodulin level, the multivariable-adjusted odds ratio for CKD was 0.6 (95% CI [0.48 to 0.81];* p* <0.01). There were no significant differences in the minor allele frequency between CKD cases and controls (*p* = 0.59) nor between first-degree relatives and controls (*p* = 0.98). There were no significant associations between genotype at rs13333226 and urine uromodulin levels (*p* = 0.43). Higher levels of urinary uromodulin are associated with lower odds of hypertension-attributed CKD. We did not detect associations of genotype at rs13333226 with urinary uromodulin levels in our sample population. Larger sample size studies from ethnically disparate populations are essential to further categorize this association.

## 1. Introduction

Tamm-Horsfall protein (THP), a mucoprotein that inhibits hemagglutination of viruses, was first discovered in the urine of healthy adults by Tamm and Horsfall in 1952 [1]. Thirty years later, uromodulin, a glycoprotein which inhibits* in-vitro* assays of human T-cell and monocyte activity was purified from urine [2]. Using characterization of complementary DNA and genomic clones, THP and uromodulin were found to be the same glycoprotein [3]. Mutations in the uromodulin (*UMOD*) gene cause autosomal dominant kidney diseases, medullary cystic kidney disease 2 (MCKD2), and familial juvenile hyperuricemic nephropathy (FJHN), which present with juvenile onset of polyuria, hyperuricemia, gout, and progressive nephropathy [4].

Uromodulin, the most abundant protein in urine [4–6], is synthesized mainly by the epithelial cells lining the thick ascending loop of Henle (TAL) [7] and also in the early part of the distal convoluted tubule [8]. Urinary uromodulin levels are reduced in patients with kidney disease [6], due to a reduction in secretion from damaged tubules [9].

The first genome wide association study (GWAS) on chronic kidney disease (CKD) identified single nucleotide polymorphisms (SNPs) in significant association with CKD at the* UMOD *locus [10]. Four loci were identified in association with eGFR_crea_, of which the strongest association was for SNP rs129177070, with the minor T allele associated with a 20% reduction in the risk of CKD [10]. In a GWAS on hypertension, the minor G allele of rs13333226 in the* UMOD *gene was associated with a lower risk of hypertension, reduced urinary uromodulin excretion, and better renal function [11].

The objective of our study was to investigate the relationship and association of urinary uromodulin levels with SNP rs1333226 in the* UMOD* gene in black South Africans

## 2. Methods

This was a case control study conducted at Charlotte Maxeke Johannesburg Academic Hospital and Chris Hani Baragwanath Academic Hospital, Gauteng Province, South Africa. Ethical clearance was granted by the Human Research Ethics Committee of the University of the Witwatersrand, South Africa (Clearance certificate no M141192), and study participants were recruited after providing written informed consent. Seventy-one unrelated black South African patients with clinically diagnosed hypertension-attributed CKD (age ≥ 18 years at disease onset) were recruited. The diagnosis of hypertension-attributed CKD was a clinical diagnosis based on typical features as assessed by the treating physician (presence of hypertension or use of antihypertensive agents, mild or no proteinuria [proteinuria ≤ 2.2 g/24h]) [12] or typical histological changes of hypertensive nephrosclerosis if a kidney biopsy was available. Patients with diabetes mellitus, other known causes of CKD, and/or seropositivity for HIV were excluded.

Patients were considered as “black” South African if they self-reported all four grandparents as being black South African. All available first-degree relatives (parents, siblings, and offspring) were included. A total of 52 first-degree relatives from 42 families were recruited, comprising 5 parents, 18 siblings, and 29 children. Also included were geographically and ethnically matched healthy controls, with normal kidney function, negative HIV serology, and normal blood pressure.

### 2.1. Clinical Parameters

Blood pressure (BP) was measured using an automated digital monitor (Rossmax PA, USA), after 5 min of rest, in the right arm and in a sitting position. Three consecutive BP readings were obtained using an appropriately sized cuff, 30-60 s apart. Hypertension was based on a history of physician diagnosed hypertension and/or receiving medications for hypertension or average systolic blood pressure ≥ 140 and/or average diastolic blood pressure ≥ 90 mmHg in adults ≥ 18 years.

Fasting serum samples for serum creatinine (using the isotope dilution mass spectrometry traceable assay) and serum uric acid were analyzed using a Cobas 6000 analyser (Roche Diagnostics, Germany). Glomerular filtration rate (GFR) was estimated using the Chronic Kidney Disease Epidemiology Collaboration (CKD-EPI) equation [13].

### 2.2. Uromodulin Measurements

Second morning urine samples were collected (mid-stream) in sterile containers and aliquoted into 1.5ml tubes immediately after collection and stored at -80°C before analysis. Chronic medications were withheld until after specimen collection. Urinary uromodulin concentrations were measured using Luminex® Performance Assay multiplex kits (R & D Systems, Inc., Minneapolis, USA). Samples were diluted 1: 4000. Fluorescence for uromodulin was read in bead region 64 on the Bio-Plex™ 200 system (Bio-Rad, Texas, USA) and concentrations were generated automatically with Bio-Plex™ manager software, version 5.0 (Bio-Rad Laboratories Inc, Hercules, USA).

### 2.3. Genomic DNA Extraction

Genomic DNA was extracted from whole blood using the salting out procedure [14] and the Maxwell® automated nucleic acid extraction platform from Promega® (Madison, USA), according to the manufacturer's instruction.

### 2.4. Polymerase Chain Reaction (PCR) Amplification and Sequence Analysis

Genotyping of rs13333226 was performed using TaqMan® SNP genotyping assays (Applied Biosystems, Foster City, USA). The sequence for the primer is as follows: GTCAAAGAGGTAGCACAGCTGTAGG [**A/G**]ATATTGACTCCTCTTCCCAAACAGC

Assays were run at a final volume of 25 *μ*L consisting of 13.75 *μ*L of TaqMan® Universal PCR Master Mix (Applied Biosystems, Foster City, USA) and 11.25 *μ*L of the sample DNA. Thermocycling conditions included an initial enzyme activation step at 95°C for 10 min and 50 cycles of denaturation at 95°C for 15 s and annealing and extension at 60°C for 60 s. All PCR reactions and allelic discrimination reactions were performed using an ABI 7500 Real-Time PCR system (ABI, Foster City, USA). Genotype clustering and calling were performed using the Sequence Detection System (SDS) software version 2.3 (ABI, Foster City, USA).

### 2.5. Statistical Analysis

Data were analyzed using STATA v12.0 (Texas, USA). The Shapiro-Wilk's test was used to test for normality resulting in uromodulin levels being modelled using a logarithmic transformation to improve normality. Continuous variables were expressed as means ± standard deviations (SD) or as medians and interquartile ranges (IQR) and discrete variables reported as percentages. Categorical data were compared using chi-square test and continuous data using Mann-Whitney, Kruskal-Wallis, analysis of variance (ANOVA) or* t*-tests, as appropriate. Logistic regression models were fitted to test for the association between the risk of kidney disease, defined as CKD-EPI eGFR < 60ml/min per 1.73 m^2^ and each outcome. The chi-square test was used to determine significant differences in allele/genotype frequencies in the* UMOD* gene between CKD cases and controls, between first-degree relatives and controls and between CKD cases and first-degree relatives. Genotype distribution was analyzed under additive genetic models using the Kruskal-Wallis test.* P* < 0.05 was considered statistically significant.

## 3. Results

### 3.1. Baseline Characteristics

We recruited a total of 181 black South African participants (71 patients with hypertension-attributed CKD, herein referred to as CKD cases, 52 first-degree relatives and 58 controls). Demographic and clinical information of the participants is shown in Table 1. There were statistically significant differences in age between CKD cases and controls (*p* < 0.001) and between first-degree relatives and controls (*p* = 0.046). Median eGFR was significantly lower in CKD cases (8 ml/min/1.73m^2^) compared to controls (121 ml/min/1.73m^2^),* p* < 0.001. However, there was no difference in the median eGFR between first-degree relatives and controls,* p *= 0.409. Cases with CKD had significantly higher systolic and diastolic BP compared to controls (*p* < 0.001), although 93% of CKD cases were on antihypertensive therapy. Among CKD cases, 81% were on loop diuretics (furosemide), while 60% were on 3 or more antihypertensive medications, which included calcium channel blockers, beta blockers, and vasodilators.

Of the first-degree relatives, 24.5% were known hypertensive, while a further 17% were diagnosed with hypertension during the recruitment period. First-degree relatives had significantly higher systolic and diastolic BP compared to controls (*p* = 0.002 and* p* = 0.006, respectively).

### 3.2. Urinary Uromodulin Levels

Urine samples were available in 166 (92%) participants (65 CKD cases, 46 first-degree relatives, and 55 controls). The mean log uromodulin (*μ*g/ml) levels were significantly lower in CKD cases (-0.4 ± 1.9) compared to controls (1.1 ± 1.7), (*p *< 0.001), as shown in Table 1. The mean log uromodulin levels were similar when comparing first-degree relatives (0.9 ± 1.2) and controls (1.1 ± 1.7), (*p *= 0.524), both of whom had normal kidney function. There were no differences in urinary creatinine levels between CKD cases and controls, with significantly lower uromodulin-to-creatinine values in CKD cases compared to controls, due to lower uromodulin levels in CKD cases.

Log uromodulin levels weakly and negatively correlated with serum uric acid levels (r_s_ = -0.33,* p* <0.001), serum creatinine (r_s_ = -0.39,* p* < 0.001), and systolic BP (r_s_ = -0.23,* p* = 0.004). There was a weakly positive correlation between log uromodulin levels and CKD EPI eGFR (r_s_ = 0.36,* p* < 0.001). Among CKD participants, there was no correlation between uromodulin levels and urinary total protein levels (r_s_ = 0.08,* p* = 0.559). For each 1-standard deviation increase in uromodulin level, the multivariable-adjusted odds ratio for CKD was 0.6 (95% CI [0.48 to 0.81];* p* < 0.001; Table 2).

### 3.3. *UMOD* SNP rs13333226

A total of 180 (99%) participants were successfully genotyped. Allele and genotype frequencies are presented in Table 1. We found no significant differences in the allele frequency (A, G) between CKD cases and controls (*p* = 0.592), between first-degree relatives and controls, (*p* = 0.983), or between CKD cases and first-degree relatives (*p* = 0.625). There were no significant differences in genotype frequencies (AA/AG/GG) between CKD cases and controls (*p* = 0.868), between first-degree relatives and controls (*p* = 0.766), or between CKD cases and first-degree relatives (*p* = 0.592).

Among CKD cases, there were no differences in systolic BP, diastolic BP, serum creatinine, and serum uric acid levels based on genotype (AA/AG/GG) (all* p* > 0.05), Table 3. For first-degree relatives, there were no differences in systolic BP, diastolic BP, serum creatinine, serum uric acid, and uromodulin levels based on genotype (AA/AG/GG), all* p *> 0.05 (Table 4).

### 3.4. SNP rs13333226 and Log Uromodulin Levels

Only the 166 participants who had both urinary uromodulin levels and genotype data were included in this analysis. Genotype at rs13333226 was not associated with uromodulin levels (*p* = 0.430). On* post hoc* analysis, among CKD cases, we found significant differences in uromodulin levels based on genotype, with cases homozygous for the ancestral A allele having higher uromodulin levels compared to those homozygous for the minor G allele (*p* = 0.05). There were no differences observed among those with the AA vs. AG or between GG vs. AG genotypes (all* p* > 0.05).

## 4. Discussion

In this study, we have demonstrated that urinary uromodulin is a marker of kidney disease, with lower levels in CKD cases compared to those with normal kidney function. We have demonstrated that urinary uromodulin correlates negatively with serum creatinine and positively with eGFR. With each 1-SD increase in urinary uromodulin, there were lower odds of CKD (OR = 0.6). The lower levels of urinary uromodulin in CKD are postulated to be secondary to a reduction in the number of functional distal tubular cells [6] and have been observed in previous case control studies on kidney disease. In a study by Thornely et al. (1985), there was a strong positive correlation between 24h uromodulin levels and creatinine clearance, regardless of the type of CKD. In participants with diabetes mellitus, urinary uromodulin levels were significantly lower in those with diabetic nephropathy compared to those with normal kidney function [15]. In IgA nephropathy, urinary uromodulin levels were lower in patients with more tubular atrophy/ interstitial fibrosis [16]. In patients with type 1 diabetes mellitus, low urinary uromodulin levels were associated with an eightfold increased risk of renal failure and cardiovascular disease [17]. Population studies showed that urinary uromodulin levels were positively associated with creatinine clearance [18]. It is postulated that uromodulin could be a marker of tubular damage or it could directly be involved in the pathogenesis of CKD. In* in vitro* studies, uromodulin resulted in interstitial inflammation and an increase in serum levels of the pro-inflammatory cytokines, TNF-*α*, IL-6, IL-8, and IL-1 ß [9]. In contrast, in* UMOD* knockout mice, uromodulin, working through toll-like receptor 4 was shown to decrease inflammation in the outer medulla [19]. Studies investigating the role of uromodulin in CKD progression in humans are necessary.

This is the first study to characterize urinary uromodulin concentrations in a black African population. Urinary uromodulin levels in our participants of black South African ancestry were lower than those previously reported among persons of European descent [18]. In persons of European descent, 24 hour urinary uromodulin levels were positively and linearly associated with renal length and volume assessed using ultrasonography, suggesting that uromodulin can be considered as a marker for tubular numbers reflected by tubular mass [18]. In earlier work, Brenner et al. suggested that reduced glomerular numbers present at birth increased the susceptibility of African Americans to diabetic nephropathy [20]. However, in an autopsy study using unbiased dissector/fractionator stereological technique to count glomeruli, the total number of glomerular numbers in African Americans was not found to be significantly lower than that in European Americans [21]. Recently, it was shown that 24h urinary uromodulin levels positively correlated with birth weight in persons of European descent, suggesting that urinary uromodulin could be used as a surrogate of nephron mass [22]. It will be important to determine the association in urinary uromodulin levels and birth weight in different ethnic groups.

In a South African study, black South Africans had lower fractional distal sodium reabsorption compared to white Belgians, possibly as compensatory mechanism as a result of enhanced proximal sodium reabsorption [23]. This suggests that there are ethnic differences in proximal and tubular functions which need to be further evaluated.

We found no difference in allele or genotype frequencies between CKD cases and controls nor between first-degree relatives and controls. Uromodulin levels were not associated with genotype at rs13333226 in the* UMOD* gene. In a study by Padmanabhan et al. (2010), the G allele of rs13333226 was associated with reduced urinary uromodulin excretion, with each copy of the G allele associated with 0.2 mg/mmol lower urinary uromodulin (*p* = 0.007). Other SNPs in the* UMOD* gene have also been associated with CKD; the C allele in rs4293393 in the* UMOD* gene was found to be protective against CKD as it was associated with lower urinary uromodulin levels and higher eGFRs [24]. We did not demonstrate an association of uromodulin levels and genotype at rs13333226 possibly because our study was conducted in black South Africans whereas participants in previous studies were predominantly white. Moreover, we had a relatively young cohort; SNPs in the* UMOD* gene have been more strongly associated with kidney function in older persons compared to younger individuals, suggesting that* UMOD* might act as a modifying factor in age-related risk factors for CKD [25].

The minor G allele at rs13333226 was previously shown to be associated with a lower risk of hypertension, with each copy of the G allele associated with 0.49 mmHg lower systolic BP and 0.30 mmHg lower diastolic BP [11]. Padmanabhan et al. (2010) used subjects with extreme hypertension, based on two BP measurements of ≥160 mmHg systolic and ≥100 mmHg diastolic BP, without any antihypertensive medication. In our study, we used less stringent BP values, with the CKD cases having relatively lower BP levels, with median (IQR) systolic and diastolic BPs of 141 (132-168) and 86 (78-97), respectively, although the 93% of CKD cases were on antihypertensive therapy. Furthermore, Padmanabhan et al. (2010) used population controls with extreme normotension (normotensive during a 10-year follow-up period). In our study, controls had BPs measured at a single-time-point.

The function of uromodulin is currently unclear. It has been postulated that it might have a role in water and electrolyte homeostasis in the TAL [5]. Uromodulin has been shown to regulate ion transport in the kidney by regulating the activities of the potassium channel ROMK [26] and the sodium-potassium-chloride transporter (NKCC2) which is necessary for active transport of sodium chloride across the TAL cells [27]. Its expression is increased by a high-salt diet and chronic use of loop diuretics [28]. In studies on* UMOD* knockout mice, it was demonstrated that* UMOD* regulates sodium uptake in the TAL by modulating the effects of tumor necrosis factor *α* on NKCC2A [29]. Uromodulin was also was shown to be protective against urinary tract infections [30] due to its ability to bind urinary tract pathogens like Escherichia coli and preventing their binding to uroplakin receptors [31]. Uromodulin plays a role in the prevention of kidney stone formation by reducing the aggregation of calcium crystals [32, 33]. In vitro studies demonstrated that uromodulin could have a role in innate immunity, by binding to immunoglobulin G, complement 1q, and tumor necrosis factor-*α* [34–36].

Our study had several limitations, which included a relatively small sample size, consisting of 181 participants. We lacked ethnic diversity as we only included participants of black South African ancestry, which constitute approximately 80% of attendees in our institution; therefore we are uncertain about the generalizability of our results to other racial/ethnic groups. We lacked 24h urine collections to better quantify uromodulin levels. We did not measure serum uromodulin level as its diagnostic utility is unclear [9].

## 5. Conclusion

In conclusion, higher levels of urinary uromodulin are associated with lower odds of hypertension-attributed CKD. We did not detect associations of genotype at rs13333226 with urinary uromodulin levels, possibly due to the young age of our participants. As* UMOD* has been implicated in kidney function and BP control, studies with larger sample sizes and from ethnically disparate populations are essential to further categorize this association.

## Figures and Tables

**Table 1:** Characteristics of the study population.

Characteristics	CKD Cases	First-degree relatives	Controls	*p*-value^*∗*^	*p*-value^#^
	(N = 71)	(N = 52)	(N = 58)		
Male, N (%)	46 (65%)	17 (33%)	26 (45%)	0.023	0.193
Age, years	48 (41-53)	30 (21-50)	41 (34-46)	<0.001	0.046
BMI, kg/m^2^	28 (24- 30)	27 (25-33)	28 (24-32)	0.634	0.853
Systolic BP, mmHg	141 (132-168)	129 (120-149)	118 (113-130)	<0.001	0.002
Diastolic BP, mmHg	86 (78-97)	76 (72-88)	73 (69-77)	<0.001	0.006
Serum creatinine, *μ*mol/L	769 (482-1116)	76 (64-84)	74 (65-83)	<0.001	0.853
Serum uric acid mmol/L,	0.5 (0.4-0.6)	0.3 (0.3- 0.3)	0.3 (0.3-0.4)	<0.001	0.105
CKD-EPI eGFR, ml/min per 1.73 m^2^	8 (4-12)	116 (91-139)	121 (99-130)	<0.001	0.409
Urine creatinine, mgdl	83 (58-120)	97 (53-134)	81 (57-140)	0.886	0.791
Uromodulin, *μ*g/ml	0.47 (0.2-3.2)	2.2 (0.9- 8)	2.8 (0.7- 9.5)	<0.001	0.866
Log Uromodulin concentration (*μ*g/ml), mean ± SD	-0.4 ±1.9	0.9 ± 1.2	1.1 ± 1.7	<0.001	0.524
u*UMOD*/uCr ratio (*μ*g/mg)	0.6 (0.2-3)	3.6 (1.2-8.1)	3 (1.0- 7.0)	<0.001	0.979
Rs13333226 genotype frequencies (AA, AG, GG), %	35, 44, 21	43, 35, 22	40, 41,19	0.868	0.766
Rs13333226 allele frequency (A, G), %	57, 43	60, 40	60, 40	0.592	0.983

Data presented as median (IQR), mean ± SD or n (%), as appropriate

^*∗*^ *p*-value: CKD cases vs. controls

^#^ *p*-value: first-degree relatives vs. controls

*P-*values from Pearson's chi square, Kruskal-Wallis, Mann-Whitney and *t*-test

Abbreviations: BMI, body mass index; BP, blood pressure; eGFR, estimated glomerular filtration rate; CKD-EPI, Chronic Kidney Disease Epidemiology Collaboration.

**Table 2:** Association with hypertension-attributed chronic kidney disease.

Characteristic	Unadjusted		Adjusted*∗*	
	Odds Ratio (95% CI)	*p*-value	Odds Ratio (95% CI)	*p*-value
*Age, Years*				
Linear Trend	1.06 (1.03 - 1.09)	<0.001	1.02 (1.00 - 1.06)	<0.001

*Sex*				
Male	1 (Ref)		1 (Ref)	
Female	0.35 (0.19 - 0.65)	0.001	0.27 (0.11 - 0.64)	<0.001

*Log Uromodulin concentration *				
Linear trend	0.6 (0.47 – 0.74)	<0.001	0.62 (0.48 – 0.81)	<0.001

*∗*Adjusted for age and sex

**Table 3:** Relationship between rs13333226 polymorphisms in the *UMOD* gene and clinical and demographic features in CKD cases.

rs13333226	AA	AG	GG	*p*-value^#^
N (%)	25	31	15	
Gender (M/F)	15/10	19/12	12/3	0.379^*∗*^
Age, years	44 (40-50)	49 (44-53)	47 (42-57)	0.157
Systolic BP, mmHg	141 (128-166)	151 (135-170)	138 (130-141)	0.327
Diastolic BP, mmHg	87 (78-97)	90 (79-100)	82 (76-87)	0.333
Serum creatinine, *µ*mol/L	823 (505-1141)	638 (513-1054)	810 (462-1015)	0.790
CKD-EPI eGFR, ml/min per 1.73 m^2^	6 (4-12)	9 (4-11)	7 (5-13)	0.833
Uric acid, mmol/L	0.4 (0.4-0.6)	0.5 (0.4-0.6)	0.5 (0.4-0.5)	0.666
Urine PCR g/mmol	0.1 (0.1-0.2)	0.1 (0.1-0.2)	0.1 (0.0-0.2)	0.315
Log Uromodulin concentration (*µ*g/ml), mean (SD)	0.3 ± 1.9	-0.5 ± 1.76	-1.4 ± 1.7	0.045^$^

Data given as median (interquartile range) unless specified.

P values: ^#^Kruskal-Wallis unless specified; ^*∗*^Pearson's chi square; ^$^ANOVA tests.

BP, blood pressure; eGFR, estimated glomerular filtration rate; CKD-EPI, Chronic Kidney Disease Epidemiology Collaboration; PCR, protein: creatinine ratio.

**Table 4:** Relationship between rs13333226 polymorphisms in the* UMOD* gene and clinical and demographic features in first-degree relatives.

rs13333226	AA	AG	GG	*p*-value^#^
N (%)	22	17	11	
Gender (M/F)	8/14	6/11	3/8	0.865^*∗*^
Age, years	34 (22-53)	29 (20-51)	25 (22-41)	0.684
Systolic BP, mmHg	126 (120-139)	135 (119-149)	128 (120-132)	0.662
Diastolic BP, mmHg	76 (72-87)	75 (72-89)	76 (69-82)	0.682
Serum creatinine, *µ*mol/L	77 (64-84)	76 (66-86)	65 (62-92)	0.601
CKD-EPI eGFR, ml/min per 1.73 m^2^	118 (82-138)	105 (91-137)	139 (87-143)	0.398
Uric acid, mmol/L	0.3 (0.3-0.4)	0.3 (0.3-0.4)	0.3 (0.2-0.3)	0.752
Urine ACR mg/mmol	1.2 (0.4-2.9)	0.4 (0.0-2.2)	2.9 (1.4-4.2)	0.225
Log Uromodulin concentration (*µ*g/ml), mean ±SD	1.0 ± 1.1	0.8 ± 1.5	1.1 ± 1.0	0.874^$^

Data given as median (interquartile range) unless specified

P values- ^#^Kruskal-Wallis unless specified; ^*∗*^Pearson's chi square; ^$^ANOVA tests

Abbreviations: BP, blood pressure; eGFR, estimated glomerular filtration rate; CKD-EPI, Chronic Kidney Disease Epidemiology Collaboration; ACR, albumin: creatinine ratio.

## Data Availability

The data used to support the findings of this study are available from the corresponding author upon request.
